# Draft Genome Sequence of Bifidobacterium pseudocatenulatum Bif4, Isolated from Healthy Infant Feces

**DOI:** 10.1128/MRA.00561-20

**Published:** 2020-06-25

**Authors:** Atul Munish Chander, Shashank Singh, Shikha Sharma, Vasvi Chaudhry, Sivasubramanian Rajarammohan, Shrikant Subhash Mantri, Mahendra Bishnoi, Sanjay Kumar Bhadada, Kanthi Kiran Kondepudi

**Affiliations:** a Healthy Gut Research Group, Food and Nutrition Biotechnology Division, National Agri-Food Biotechnology Institute, Mohali, Punjab, India; b Department of Microbial Interactions, IMIT/ZMBP, University of Tuebingen, Tuebingen, Germany; c Department of Endocrinology, Postgraduate Institute of Medical Education and Research, Chandigarh, India; University of Rochester School of Medicine and Dentistry

## Abstract

We report the 2.24-Mb draft genome sequence of Bifidobacterium pseudocatenulatum Bif4, isolated from a fecal sample from a healthy infant. The specific annotations revealed genes predictive of its probiotic attributes.

## ANNOUNCEMENT

Bifidobacterium pseudocatenulatum Bif4, which has an ability to reduce lipopolysaccharide-induced inflammation in murine macrophages, was isolated from a fecal sample from a healthy infant.

The study was approved by the Institutional Ethics Committee of the Postgraduate Institute of Medical Education and Research (PGIMER) in Chandigarh, India. The fecal sample was collected from the infant after an informed consent form was signed by the parent. The sample was homogenized in sterile phosphate-buffered saline containing 0.05% l-cysteine (pH 7.4, 0.0018 M), serially diluted, and spread onto De Man, Rogosa, and Sharpe agar plates containing filter-sterilized 0.05% (wt/vol) each of l-cysteine hydrochloride (MRSC) and 0.05% mupirocin (Hi-Media, India) (1) followed by incubation at 37°C for 48 h under obligate anaerobic conditions (80% N_2_ + 10% CO_2_ + 10% H_2_) using an Anoxomat culture system (Mart, Lichtenvoorde, Netherlands). A single colony of the strain was subcultured three times on MRSC agar plates every 48 hours. Genomic DNA was extracted from 24-hour-grown culture on MRSC agar plates using a ZR fungal/bacterial DNA miniprep kit as per the manufacturer’s instructions. Genomic DNA (1.0 μg) was used as input material for library preparation. The sequencing library was generated using a TruSeq Nano DNA high-throughput sample preparation kit (Illumina, USA) following the manufacturer’s recommendations. The genomic DNA was randomly fragmented to a size of 350 bp using Covaris cracker, and DNA fragments were then end polished, A tailed, and ligated with the full-length adapter for Illumina sequencing with further PCR amplification. The amplicons obtained were purified (AMPure XP system, Beckman Coulter), and libraries were analyzed for size distribution using an Agilent 2100 bioanalyzer and quantified using real-time PCR. Paired-end sequencing was performed using an SBS kit v4 on an Illumina HiSeq 2500 sequencing platform.

Default parameters for all software were used in this study unless otherwise specified. Before assembly, raw data were passed into Trimmomatic v0.36 for trimming adapters, low-quality bases, and stretches of sequence ambiguities (N, bases that cannot be determined) using a sliding window filter. Thus, reads containing adapters, reads containing >10% N, and reads containing low-quality bases (Q score, ≤5) were removed, which was over 50% of the total bases. Genome assembly was performed with CLC Genomics Workbench (CLC NGS Cell v2018). The total number of reads was 32,738,540 with an average read length of 141.21 bp; of these, 31,890,222 reads were in pairs with an average read length of 339.68 bp. The assembled genome was 2,214,245 bp long with a GC content of 56.50%. The assembled genome contained 58 contigs with an *N*_50_ value of 272,576 bp, *L*_50_ of 4, and genome coverage of 2,061.0×. The average contig length was 25,478 bp, and the largest contig was 418,722 bp. The genome of Bif4 was 100% complete and contained 0.45% sequence contamination as determined using CheckM v1.0.18 (2).

Genome annotations were assessed with Rapid Annotations using Subsystems Technology (RAST) and the NCBI Prokaryotic Genome Annotation Pipeline (PGAP). PGAP annotation identified 1,841 total genes, 1,781 total coding DNA sequences, 60 RNA genes, and 52 tRNAs. Genome assembly and its PGAP annotation are publicly available. RAST annotated the genome into 265 subsystems containing a total of 996 proteins (3). CRISPRCasFinder identified a total of nine CRISPR sequences in various contigs of the genome (4). EzBioCloud revealed that the 16S rRNA gene sequence of Bif4 had the highest identity (99.09%) with Bifidobacterium pseudocatenulatum DSM 20438^T^ and 98.39% identity with Bifidobacterium catenulatum subsp. *catenulatum* DSM 16992^T^ (5). The average nucleotide identity (ANI) value of Bif4 was calculated using the ANI calculator with closely related genomes (6). Results suggested that Bif4 shared ANI values of 97.43% and 82.41% with Bifidobacterium pseudocatenulatum DSM 20438^T^ and Bifidobacterium adolescentis ATCC 15703^T^, respectively. This further indicated its species assignment to Bifidobacterium pseudocatenulatum with a ≥95% cutoff value for species delineation (5).

Bif4 harbors 41 genes responsible for central carbohydrate metabolism, including 8 genes coding for fructooligosaccharide and raffinose utilization, 9 genes for lactose and galactose uptake and utilization, and 15 genes for maltose and maltodextrin utilization. Importantly, the strain also contains genes required for sucrose utilization, trehalose uptake and utilization, d-ribose utilization, l-arabinose utilization, xylose utilization, glycogen metabolism, and sialic acid metabolism. The Resistance Gene Identifier tool has validated that the rifamycin-resistant beta-subunit of RNA polymerase (*rpoB*) (98.4% identity) and elfamycin-resistant EF-Tu (71.32% identity) were reported in the draft genome of Bif4, whereas the identity score was less than 60% for the other genes contributing to drug resistance (7). Functional comparison in RAST annotation revealed that Bif4 was closely related to Bifidobacterium adolescentis ATCC 15703, Bifidobacterium dentium Bd1, Bifidobacterium pseudocatenulatum DSM 20438, and Bifidobacterium catenulatum DSM 16992.

### Data availability.

The assembly (accession no. GCA_011008535.1) and draft genome sequence of *B. pseudocatenulatum* Bif4 are available as a whole-genome shotgun (WGS) project in GenBank under accession no. QCZO00000000. This is the first version of the project, QCZO01000000, and consists of sequences QCZO01000001 to QCZO01000058. The raw sequencing reads have been deposited under SRA accession no. SRP252338.
